# Data in support of the proteomic analysis of plasma membrane and tonoplast from the leaves of mangrove plant *Avicennia officinalis*

**DOI:** 10.1016/j.dib.2015.10.016

**Published:** 2015-10-26

**Authors:** Pannaga Krishnamurthy, Xing Fei Tan, Teck Kwang Lim, Tit-Meng Lim, Prakash P. Kumar, Chiang-Shiong Loh, Qingsong Lin

**Affiliations:** a Department of Biological Sciences, National University of Singapore, 14 Science Drive 4, 117543, Singapore; b NUS Environmental Research Institute (NERI), National University of Singapore, Singapore

**Keywords:** *Avicennia*, Salt tolerance, Salt secretion, Tonoplast, Plasma membrane, Proteomics

## Abstract

The data provides information in support of the research article, Proteomics 2014, 14, 2545–2557 [1]. Raw data is available from the ProteomeXchange Consortium via the PRIDE partnerRepository [2] with the dataset identifier PXD000837. Plasma membrane and tonoplast proteins from the leaves of *Avicennia officinalis* were identified using gel electrophoresis (one and two dimensional) combined with LC–MS analysis. Based on GO annotation, identified proteins were predicted to be involved in various biological processes.

**Specifications Table**

**Table:** 

Subject area	*Biology*
More specific subject area	*Membrane proteomics and mass spectrometry (MS)*
Type of data	*MS data and annotations*
How data was acquired	*Mass spectrometry: Data was acquired on the instrument TripleTOF* 5600 *(SCIEX, Foster City, CA, USA).*
Data format	*Raw and processed data*
Experimental factors	*Plasma membrane and tonoplast membrane protein fractions were isolated from the leaves of field-growing Avicennia officinalis trees using two-aqueous-phase partitioning and density gradient centrifugation, respectively.*
Experimental features	*The plasma membrane and tonoplast membrane proteins were fractionated by* 1*DE and* 2*DE. The peptides resulting from in-gel tryptic digestion were fractionated and analyzed using LC–MS/MS.*
Data source location	*Singapore*
Data accessibility	*Deposited to the ProteomeXchange Consortium via the PRIDE partnerRepository*[2]*with the dataset identifier PXD*000837 *(**http://proteomecentral.proteomexchange.org/cgi/GetDataset?ID=PXD*000837*)*

**Value of the data**

• *Avicennia officinalis is a salt secreting mangrove plant that exhibits salt tolerance to high salinities.*
• *To our knowledge, this represents the first dataset on the membrane proteomics of a mangrove plant.*
• *A total of* 254 *plasma membrane and* 165 *tonoplast proteins were identified.*
• *This data would be valuable in understanding the mechanism of salt secretion in mangroves and salt tolerance in plants.*

## 1 Experimental design

**Fig. 1:**
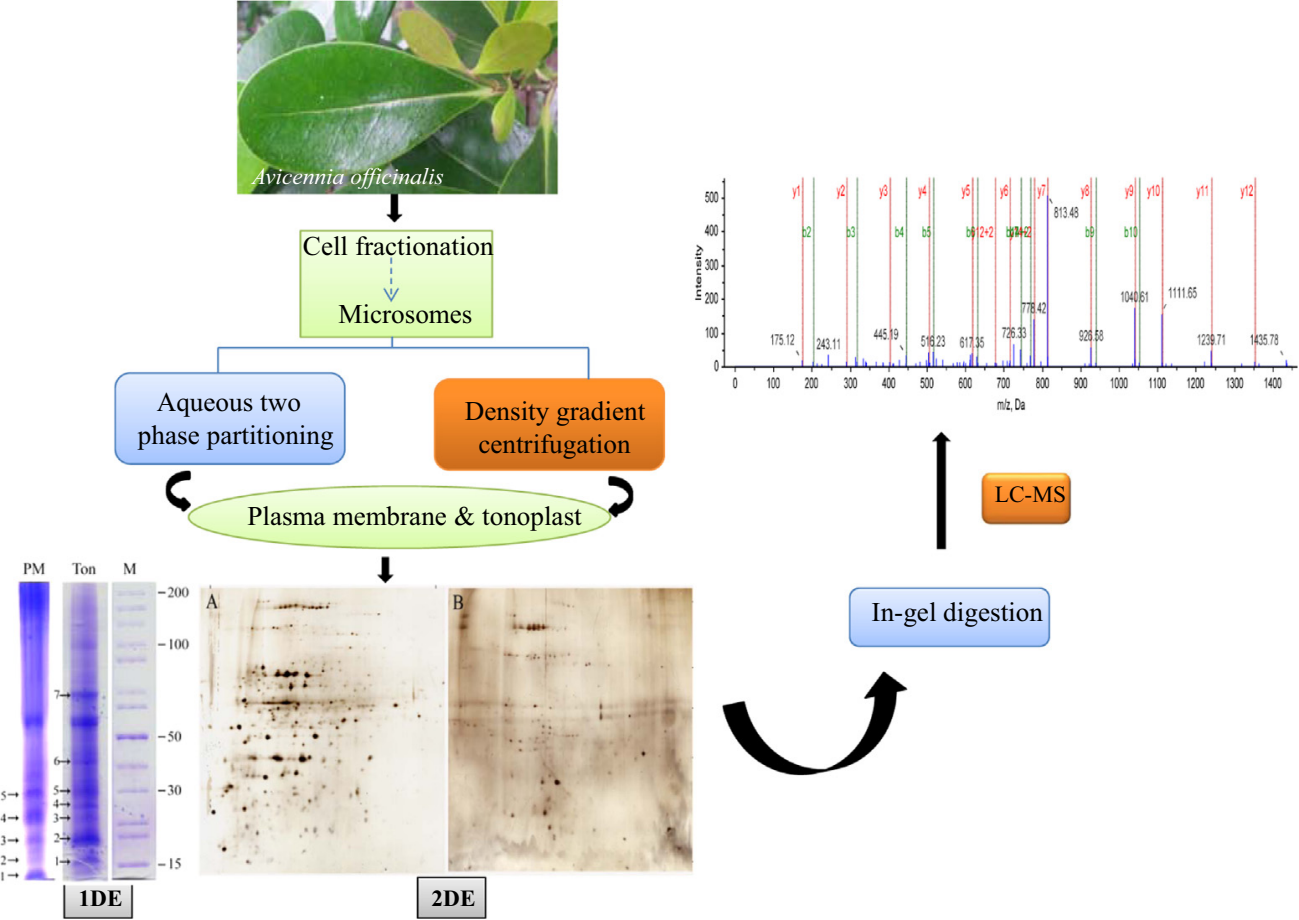
Experimental design of the membrane proteomic analysis.

Fig. 1 shows the general workflow of the membrane proteomic analysis. Leaves were collected from the mangrove trees and plasma membrane fraction was isolated using an aqueous two-phase partitioning method while the tonoplast fraction was isolated by density gradient centrifugation. The membrane proteins were fractionated by one- and two-dimensional gel electrophoresis. The trypsin digested fragments from the gels were used for LC–MS/MS analysis. MS data was acquired using a TripleTOF 5600 system and the peptide identification was carried out using the ProteinPilot 4.5 software.

**Fig. 2:**
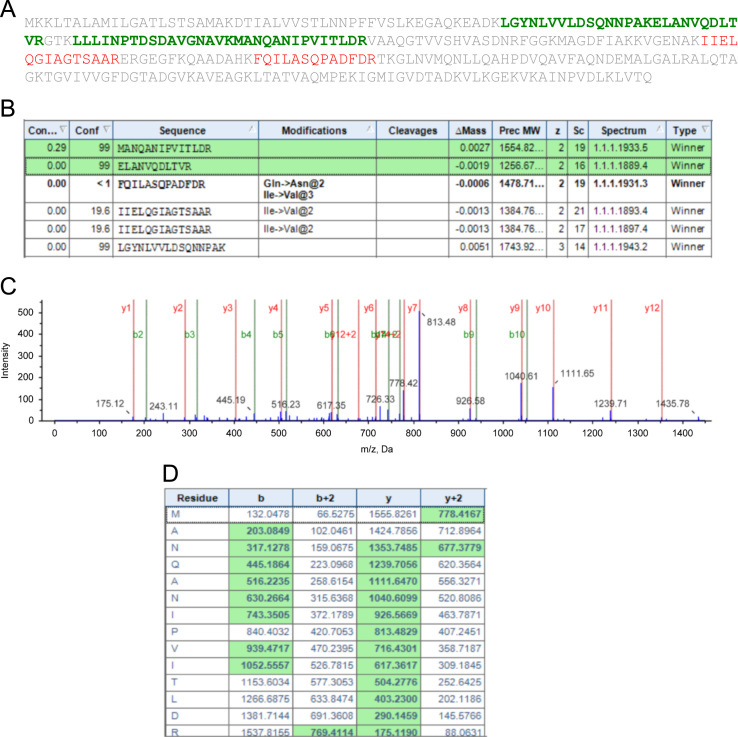
MS/MS Data analysis.

Fig. 2 shows the representative protein sequence identified with confidence interval ≥95% in the study. (**A**) Protein sequence with identified peptide sequences highlighted in green. (**B**) Table with details of identified peptides. (**C**) MS/MS spectrum of the identified peptide **MANQANIPVITLDR**. (**D**) Assignment of fragmentation for the identified peptide.

**Fig. 3:**
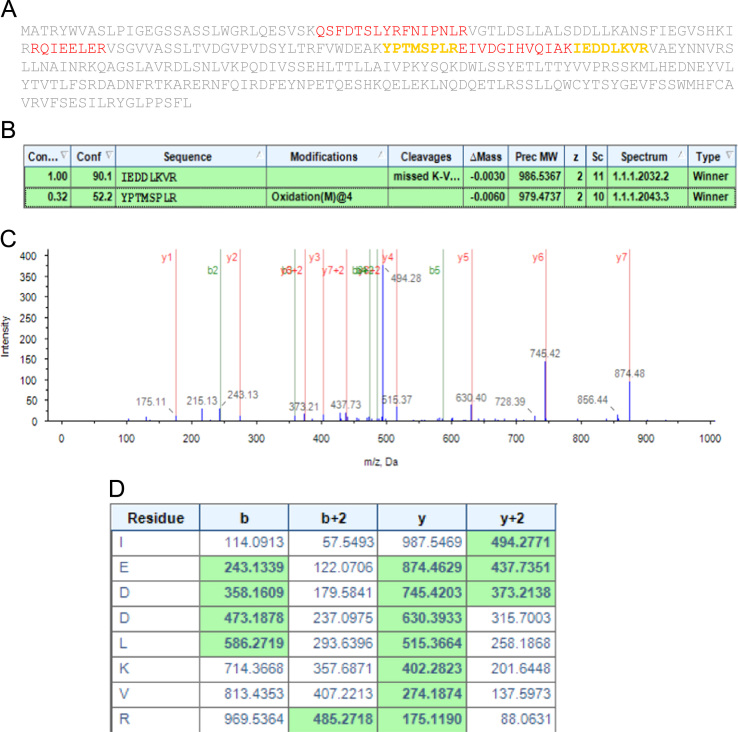
MS/MS Data analysis.

Fig. 3 shows the representative protein sequence identified with confidence level <95% but ≥50% in the study. (**A**) Protein sequence with identified peptide sequences highlighted in yellow. (**B**) Table with details of identified peptides. (**C**) MS/MS spectrum of the identified peptide **IEDDLKVR**. (**D**) Assignment of fragmentation for the identified peptide.

## 2 Materials and methods

### 2.1 Membrane preparation

Leaves were collected from the *Avicennia officinalis* trees growing in the equatorial mangrove swamps in Singapore. Plasma membrane and tonoplast fractions were prepared from freshly harvested leaves (~100 g) using two-phase partitioning [3] and density gradient centrifugation [4] methods, respectively. Briefly, to isolate PM, freshly harvested leaves were rinsed with ice-cold distilled water and homogenized in a blender using pre-chilled homogenization medium consisting of 50 mM Tris, 500 mM sucrose, 10% glycerol, 20 mM EGTA, 20 mM EDTA, 5 mM β-glycerophosphate, 1 mM phenantroline, 0.6% PVP, 10 mM ascorbic acid, 1 mM PMSF, protease inhibitor tablets (Roche), 1 mM leupeptin, 5 mM DTT and 1 mM Na-orthovanadate after adjusting to pH 8.0 with MES. The resulting homogenate was filtered through a nylon cloth (100 µm) and centrifuged at 26,000 g for 25 min at 4 °C. The resulting supernatant was centrifuged at 84,000 g for 30 min at 4 °C to obtain a microsomal pellet. The pellet was resuspended in microsomal buffer consisting of 5 mM phosphate buffer pH 7.8, 330 mM sucrose and 2 mM DTT. The microsomal fraction was further purified using a dextran – polyethylene glycol (PEG) two-phase system consisting of 20% dextran T – 500, 6.4% PEG, 5 mM phosphate buffer pH 7.8, 5 mM KCl and 300 mM sucrose. The upper phase was recovered and partitioned with fresh lower phase twice. The final upper phase was diluted at least two-fold with plasma membrane washing buffer consisting of 10 mM Tris, 10 mM boric acid, 300 mM sucrose, 9 mM KCl, 5 mM EDTA, and 5 mM EGTA and was centrifuged at 176,000 g for 30 min at 4 °C to obtain a PM-enriched pellet.

For the isolation of tonoplast, freshly harvested leaves were rinsed with ice-cold distilled water and homogenized in a pre-chilled grinding medium consisting of 0.25 M sorbitol, 5 mM EGTA, 1 mM PMSF, 2.5 mM potassium metabisulfite, 1.5% (w/v) PVP, 50 mM MOPS-KOH pH 7.6, 10 mM β-glycerophosphate, 0.45 mM butylated hydroxytoluene, protease inhibitor tablets (Roche), 5 mM DTT and 1 mM Na-orthovanadate. The tissue homogenate was filtered through 4 layers of gauze. The filtrate was centrifuged at 3600 g for 15 min at 4 °C. The resulting supernatant was centrifuged at 150,000 g for 40 min at 4 °C. The pellet was re-suspended in a buffer containing 15% sucrose (w/v), 10 mM potassium phosphate pH 7.8, 1 mM EGTA and 2 mM DTT. This suspension was overlaid with a buffer containing 0.25 M sorbitol, 5 mM MOPS-KOH pH 7.3, 1 mM EGTA and 1 mM DTT and centrifuged at 120,000 g for 1 h at 4 °C. The tonoplast membranes at the interface between sucrose and sorbitol layers were collected and diluted ~5-fold with a buffer containing 5 mM MOPS-KOH pH 7.3, 0.25 M sorbitol, 1 mM EGTA and 1 mM DTT. The suspension was centrifuged at 150,000 g for 30 min at 4 °C to obtain a tonoplast enriched pellet.

All membrane fractions were carbonate washed, following the method described by [5] to remove the soluble proteins and were stored at −80 °C.

### 2.2 One- and two-dimensional polyacrylamide gel electrophoresis (1DE and 2DE)

For 1DE, samples containing 5–10 µg of purified PM and tonoplast proteins in gel loading buffer (62.5 mM Tris adjusted to pH 6.8 with HCl, 2% (m/v) SDS, 0.1 M DTT, 10% (v/v) glycerol, 0.1% (m/v) bromophenol blue) were loaded on to precast, 4–12% gradient gels (Nu PAGE, Invitrogen). The SDS-PAGE was carried out at a constant voltage of 200 V for ~1 h. After the completion of separation, the protein bands were visualized by staining with coomassie brilliant blue and/or silver. Three independent experiments were carried out for both PM and tonoplast fractions from three biological replicates. The bands that appeared consistently in all biological replicates were selected for MS/MS analysis.

For 2DE, the PM and tonoplast pellets were resuspended in rehydration solution consisting of 7 M urea, 2 M thiourea, 4% CHAPS, 40 mM DTT, and 0.002% w/v bromophenol blue, vortexed followed by a 1 h incubation at room temperature. The supernatant consisting of solubilized PM and tonoplast proteins was then collected by centrifugation at 14,000 g for 30 min. For the first-dimension electrophoresis, 17-cm long pH 4–7 ReadyStrip IPG strips (Bio-Rad, Hercules, CA) were passively rehydrated overnight at room temperature with 340 µL of rehydration buffer containing 50 µg protein and 0.5% v/v pH 4–7 carrier ampholytes. IEF was carried out in a PROTEAN IEF cell (Bio-Rad) at a current limit of 50 µA per IPG strip at 20 °C for a total of 37.55 kV h. Each focused IPG strip was equilibrated by soaking, with mild stirring for 15 min in 10 mL of equilibration buffer 1 consisting of 6 M urea, 0.05 M Tris pH 8.8, 2% w/v SDS, 20% v/v glycerol, 2% w/v DTT followed by soaking in 10 mL of equilibration buffer 2 (same content as equilibration buffer 1 except DTT was replaced with iodoacetamide) for 15 min. The second dimension of 2DE was carried out by placing the IPG strips on to a separating gel (12% polyacrylamide, w/v). Gel electrophoresis was performed at 30 mA per gel with circulating cooling and was completed in 5 h. Protein spots were visualized by staining with silver and gel image was captured. All 2DE analyses for both PM and tonoplast fractions were carried out in three biological replicates, and spots that appeared consistently in all biological replicates were selected for MS/MS analysis.

### 2.3 In-gel digestion

The proteins were digested in-gel using MS-grade Trypsin Gold (Promega) according to the manufacturer׳s instructions. The selected bands from 1DE and spots from 2DE were carefully excised from the gels using a clean razor blade and incubated at 4 °C for 24 h in a washing buffer containing 2.5 mM NH_4_HCO_3_ and 50% ACN followed by incubation of the samples in fresh washing buffer at 37 °C for 10 min with constant shaking. Later, the samples were dried in a vacuum centrifuge (SpeedVac). Reduction of samples were carried out using 10 mM DTT followed by alkylation with 55 mM iodoacetamide. Later, alternative washing with 100 mM NH_4_HCO_3_ and dehydration using ACN was carried out. Finally, the samples were vacuum dried and trypsin digested by preincubating in 10–20 µL trypsin (0.01 µg/µL) solution at 4 °C for 30 min followed by an incubation at 37 °C for 16 h.

### 2.4 LC–MS/MS analysis

The analysis was carried out as described earlier [1]. Eksigent nanoLC Ultra and ChiPLC nanoflex (Eksigent, Dublin, CA, USA) in trap-elute configuration were used to separate the digested peptides. Sep-Pak C18 Elution Plate (Waters, Milford, MA, USA) was used to desalt the digested samples. The desalted samples were reconstituted with 15 µL of diluent (2% ACN, 0.1% formic acid (FA)). A total of 10 µL of the sample was loaded onto a 200 m×0.5 mm ChromXP C18-CL trap column and eluted onto a 75 µm×150 mm ChromXP C18-CL analytical column. Peptides were separated by a gradient formed by 2% ACN, 0.1% FA (mobile phase A) and 98% ACN, 0.1% FA (mobile phase B): In 5–7% of mobile phase B for 0.1 min, in 7–30% of mobile phase B for 10 min, in 30–60% of mobile phase B for 4 min, in 60–90% of mobile phase B for 1 min, kept at 90% of mobile phase B for 5 min, 90–5% of mobile phase B for 0.1 min, and maintained at 5% mobile phase B for 10 min, at a flow rate of 300 nL/min. TripleTOF 5600 system (SCIEX, Foster City, CA, USA) in information-dependent mode was used to perform MS analysis. MS spectra were acquired across the mass range of 350–1250 m/z in high-resolution mode (>30,000) using 250 ms accumulation time per spectrum. A maximum of 20 precursors per cycle were chosen for fragmentation from each MS spectrum with 100 ms minimum accumulation time set for each precursor and dynamic exclusion for 8 s. Tandem mass spectra were recorded in high-sensitivity mode (resolution >15,000) with rolling collision energy on. ProteinPilot 4.5 software Revision 1656 (SCIEX) was used for peptide using the Paragon database search algorithm (4.5.0.0.). The data with all the MS/MS spectra were searched against several databases, including databases of Swiss-Prot, Plant Reference Sequences (total 248,485 entries) and a database consisting of all the available mangrove plant protein sequences in NCBI. Only the proteins with highest coverage were retained while the redundant proteins identified from each band/spot were filtered. The search parameters were as follows: Sample Type—Identification; Cys Alkylation—Iodoacetamide; Digestion—trypsin; Special Factors—Phosphorylation emphasis; Species—None. The processing was specified as follows: ID Focus—Amino Acid Substitutions; Search Effort— Thorough; Detected Protein Threshold—0.05. No FDR analysis was performed on the dataset. Instead, peptides identified with confidence interval ≥95% (green color coded peptides as shown in Fig. 2) were taken into account. In addition, peptides identified with confidence level <95% but ≥50% (yellow color coded peptides as shown in Fig. 3) were manually inspected and only those were included in which all the Y ions and majority of the intense peaks were assigned. The plasma membrane and tonoplast proteins identified in the study are listed in Supplementary Tables 1 and 2 and the index to Spot/band numbering and PRIDE numbering is given in Supplementary Table 3.
