# Supplementary material for: Data in support of the proteomic analysis of plasma membrane and tonoplast from the leaves of mangrove plant Avicennia officinalis
Source: Data Brief. 2015 Oct 26;5:646–52. doi: 10.1016/j.dib.2015.10.016 (PMC4644245; doi:10.1016/j.dib.2015.10.016)
Supplement: Supplementary file 2 — Supplementary material [file mmc2.pdf]

Supporting information for online publication

Supplementary Table 1:

Plasma membrane proteins identified by Mass spectrometry

Proteins are grouped according to functional similarity. GO annotation was carried out using the STRAP software (http://www.bumc.bu.edu/cardiovascularproteomics/cpctools/strap/). Protein ID is given in column 1. Together with commonly used abbreviations, if available, the protein names are given as in uniprot/ (STRAP) in column 2 and 3. In column 4 and 5, the theoretical and experimental (Thr/Exp) molecular weight and p*I* respectively are given. The theoretical molecular weight and p*I* were calculated using ExPASy (http://web.expasy.org/compute_pi/). The % coverage is given in 6 and band / spot numbers are indicated in the column 7.

Protein ID	Protein description	protein Name	MW (kDa) Thr/Exp	p*I* Thr/Exp		%cov	band/spot index
**Transport**							
Q39196	Probable aquaporin PIP1-4	PIP1-4	30.7/30.0	9.0	6	3.5	4, 5, 58
A9CTP9	Tonoplast intrinsic protein	TIP1-1	25.5/20.0	6.20	7	4.1	3-5, 47, 58
D4IA27	Outer membrane protein A	ompA	38.3/96.0	6.8/5.6	0	3.4	59, 60
E1SJC2	ABC-type sugar transport system	rbsB	30.4/13.0	8.61	0	29.2	1, 2
Q9LVM1	ABC transporter B family member 25	ABCB25	80.4/20.0	9.3/4.1	5	4.3	11, 18
K4KCZ8	Ycf1 (Fragment)	ycf1	94.2/38.0	9.5/5.2	6	2.6	24
P02925	D-ribose-binding periplasmic protein	rbsB	30.9/13.0	6.85	0	43.2	1, 2, 5
P0AEE7	D-galactose-binding periplasmic protein		35.7/17.0	5.68	1	13.9	1, 2, 5
G8LMD4	Glucose-specific phosphotransferase enzyme IIA component	crrE	18.2/17.0	4.73	0	16.6	2, 5, 58, 60
Q36600	ATPase subunit I		19.0/20.0	9.62	0	23.5	3
K4K963	ATP synthase subunit alpha	atpA	55.1/62.0	5.7/4.7	0	19.7	42, 43, 47, 57- 60
G8E8W2	ATP synthase subunit alpha	atp1	55.5/16.5	5.7/4.2	0	8.4	6
F6HFL0	ATP synthase subunit beta		53.8/96.0	5.1/5.4	0	46.6	23, 45, 58-60, 13, 41
B9R8F3	ATP synthase subunit beta		46.3/78.0	5.3/4.7	0	32	52, 53, 54, 56, 57
P32980	ATP synthase delta chain	ATPD	26.9/20.0	6.9/4.1	0	14.2	11, 32

Q01908	ATP synthase gamma chain 1		40.9/48.0	8.1/4.7	0	3.5	26, 35
Q8M8Y9	ATP synthase epsilon chain	atpE	14.4/23	5.8/4.9	0	30.3	8, 2
Q5FRW7	ATP synthase subunit b	atpF	20.2/58.0	9.6/5.0	2	12.4	45, 2
Q2V3P9	ATP synthase subunit d	ATPQ	13.8/31.0	5.5/5.2	0	9.8	20
B9SUN3	ATP synthase D chain		19.7/31.0	5.5/5.2	0	11.9	18, 20, 25
P09469	V-type proton ATPase catalytic subunit A		68.7/48.0	5.2/4.7	0	1.3	35, 38
O23654	V-type proton ATPase catalytic subunit A	VHA-A	68.8/98.0	5.1/5.2	0	3.1	35, 38, 52, 58
Q9SZN1	V-type proton ATPase subunit B2	VHA-B2	54.3/98.0	5.0/5.2	0	20.7	53, 56- 58
Q8W4E2	V-type proton ATPase subunit B3	VHA-B3	54.3/96.0	4.9/5.4	0	9.7	52, 59
D7SH58	V-type proton ATPase subunit C		36.5/18.5	5.8/5.4	0	16.4	10
A5B4N7	V-type proton ATPase subunit F		14.5/17.0	7.99	0	12.9	2
K7S1W9	Potassium channel AKT1	AKT1	9.3/86.0	9.1/6.3	1	12.8	61
Q6L5I5	Voltage-dependent anion channel 2	VDAC2	29.6/30.0	8.56	0	3.6	5
A9CM22	Voltage-dependent anion channel 3	VDAC3	29.7/30.0	8.78	0	9.4	5
F6GU55	ADP, ATP carrier protein		42.2/30.0	9.81	3	14.3	5
P40941	ADP, ATP carrier protein 2	AAC2	20.6/30.0	4.84	0	10.7	5
Q93XM7	Mitochondrial carnitine/ acylcarnitine carrier-like protein		31.0/30.0	9.56	0	17.3	5
F4KG18	Triose phosphate/ phosphate translocator	TPT	45.2/30.0	9.67	9	4.3	5
Q8LSZ0	Betaine/proline transporter		48.0/38.0	7.2/4.8	11	3.9	23, 43
D7STS6	Proline transporter	55.6/20.0	8.4/4.1	0	3.7	11	
G8LMD2	Phosphocarrier protein HPr	ptsH	9.1/96.0	5.6/5.6	0	15.3	58- 60
G8LJ41	Leu/Ile/Val-binding protein	livJ	39.4/96.0	6.2/5.4	0	7.0	59
Q9SX98	Lysine histidine transporter-like 8	AATL1	57.1/78.0	9.4/4.7	10	2.7	53
Q5PGU4	Acyl carrier protein (ACP)	acpP	8.6/17.0	3.98	0	32.1	1, 2, 5, 58
A5BX41	Cytochrome b6-f complex iron-sulfur subunit		24.1/31.0	7.6/5.2	1	57.5	3, 17, 20, 21
E1SKK0	Phosphoenolpyruvate-protein phosphotransferase		63.1/98.0	4.9/5.2	0	10.3	58
Q9AXF3	Non-specific lipid-transfer protein		11.8/13.0	9.5	0	9.6	1, 2
A9PE72	Uncharacterized protein		22.6/26.5	5.0/4.3	0	28.6	2, 11, 13, 16
**Metabolic process**							
G8LLY7	Transaldolase		tal	35.2/13.0	5.0	2.8	1

Q9FVH1	Transaldolase	TAL	48.5/60.0	5.3/5.5	11.7	48
B9GZ15	Transaldolase family protein		9.7/56.0	4.8/5.3	12.6	45- 47
G8LGB5	KHG/KDPG aldolase		23.6/30.0	5.93	5.8	1, 5
Q9LYR4	Aldolase-type TIM barrel family protein		47.7/60.0	6.1/5.5	7.8	44- 48
G8FMI9	Fructose-bisphosphate aldolase		49.1/98.0	7.2/5.2	19.6	58
U5KP25	Fructose-bisphosphate aldolase		42.0/76.0	6.4/5.9	8.0	50
G8LDD8	Fructose-bisphosphate aldolase class 2	baA	39.1/13.0	5.6	4.7	1, 2, 5
G8LF07	Citrate synthase		48.1/13.0	5.9	2.6	1, 2, 5, 58- 60
G0ACV1	Argininosuccinate synthase	argG	49.4/96.0	5.3/5.4	4.7	59, 60
Q0RF70	Chorismate synthase		41.1/72.0	6.3/5.9	2.3	49
Q5FUB1	1-deoxy-D-xylulose-5-phosphate synthase		72.2/60.0	6.1/5.5	9.3	45, 46, 48
Q84UC3	Sucrose synthase		47.1/60.0	6.2/5.5	4.9	48
Q4KAW9	Methionine synthase family protein,					
	Vitamin-B12 independent		38.4/58.0	5.3/5.0	22.6	45, 46, 49
G8LM14	Carbamoyl-phosphate synthase large chain	carB	117.9/98.0	5.1/5.2	3.5	58
E1SKJ7	Cysteine synthase A	cysK	34.4/98.0	5.7/5.2	16.2	58, 60
E1SK88	Isocitrate dehydrogenase	icdA	45.8/96.0	5.1/5.4	10.6	1, 2, 5, 59
Q3YX11	Malate dehydrogenase	mdh	32.3/13.0	5.6	24.4	1, 2, 5, 26, 58- 60
E0CUS6	Probable NADH dehydrogenase		20.1/36.0	4.8/4.5	12.9	22
Q9XQP4	NAD-malate dehydrogenase		43.5/13.0	7.0	14.4	1, 21, 26, 58
P25553	Lactaldehyde dehydrogenase		52.3/13.0	5.1	7.1	1, 2, 5
P37769	2-dehydro-3-deoxy-D-gluconate 5-dehydrogenase		27.1/13.0	5.2	7.9	1, 2, 5, 59
Q7KW39	Probable methylmalonate-semialdehyde dehydrogenase		55.9/20.0	8.56	2.1	3
A8IJK7	Putative succinate-semialdehyde dehydrogenase		53.1/72.0	5.7/5.9	6.3	46, 48, 49
Q3KCR9	Short-chain dehydrogenase/reductase	SDR	30.7/58.0	4.9/5.2	7.4	46
G0BJS3	6-phosphogluconate dehydrogenase, decarboxylating		51.5/96.0	5.1/5.6	9.6	60
G8LKG3	Glyceraldehyde-3-phosphate dehydrogenase A	gapA	35.9/96.0	6.3/5.4	15.3	1- 3, 59, 60
Q9SAJ6	Glyceraldehyde 3-phosphate dehydrogenase	GAP	17.498.0	7.8/5.2	9.0	47, 58
E0SN64	Inosine-5'-monophosphate dehydrogenase		51.7/98.0	6.8/5.2	5.7	58
E1SCY6	D-3-phosphoglycerate dehydrogenase	serA	44.1/98.0	5.9/5.2	7.3	58
G8LJ17	Aspartate-semialdehyde dehydrogenase	asd	39.9/98.0	5.4/5.2	4.9	58-60
G8LMU0	Pyruvate dehydrogenase E1 component		99.6/96.0	5.5/5.6	4.1	60
G9IBB5	NAD(P)H-quinone oxidoreductase subunit 4L	ndhE	11.3/17.0	9.74	8.9	2
A9PC01	NADH2 dehydrogenase family protein		12.2/17.0	7.86	11.4	2

G8LDD9	Phosphoglycerate kinase	pgk	30.4/13.0	5.1	7.2	1, 2, 5, 58- 60
Q7UB84	Glycerol kinase	glpK	56.2/13.0	5.3	15.9	1, 2, 5
Q2JL49	Glycerate kinase		39.5/96.0	4.9/5.0	4.2	57
G8LID3	Ribose 1,5-bisphosphate phosphokinase	phnN	20.5/16.5	9.3/4.2	6	6
Q9KNK0	Phosphoenolpyruvate carboxykinase	pckA	59.8/13.0	5.6	3.1	1
G8LCJ8	Enolase	eno	47.4/96.0	5.1/5.6	7.4	1, 2, 5, 58- 60
G8LHG4	Isocitrate lyase		48.2/96.0	5.4/5.6	4.1	2, 60
Q4VZH5	Cytochrome b559 subunit alpha	psbE	9.4/13.0	4.8	48.2	1, 2, 5
Q4VZK4	Cytochrome b6-f complex subunit 4		17.4/17.0	6.6	10.0	2
Q3KEU6	Putative isochorismatase family protein		22.8/17.0	5.2	9.1	2
Q5PCM7	Succinyl-CoA ligase [ADP-forming]	sucC	41.5/17.0	5.3	9.5	2
G8LF15	Succinyl-CoA ligase [ADP-forming]	sucD	29.8/30.0	6.09	7.6	1, 5, 59
Q8FHG5	Glutamate decarboxylase beta	gadB	52.6/17.0	5.35	18.9	1, 2, 5
P0C1I2	Peptidyl-prolyl cis-trans isomerase E		34.7/20.0	5.53	5.1	3, 4
B1YFD1	Peptidyl-Prolyl cis trans isomerase		4.1/48.0	4.7/4.7	5.3	35, 28
G8LKY0	Ketol-acid reductoisomerase	ilvC	54.1/98.0	5.2/5.2	19.4	46, 50, 57- 60
G8LL70	Phosphopentomutase		26.5/30.0	6.91	2.9	5
Q8XA55	Serine hydroxymethyltransferase (SHMT)	glyA	45.3/30.0	6.03	5.8	2, 5, 45, 60
E0SEA3	5-methyltetrahydropteroyltriglutamate-					
	-homocysteine methyltransferase		42.8/58.0	6.3/5.2	24.4	46, 49
G8LK46	5-methyltetrahydropteroyltriglutamate-					
	-homocysteine methyltransferase	metE	84.7/98.0	5.8/5.2	13.2	43, 50, 58, 59, 60
B3PIH6	UTP-glucose-1-phosphate uridylyltransferase		31.5/60.0	5.8/5.5	6.4	23, 44- 49
G8LNR6	Putative methylcobalamin: homocysteine					
	methyltransferase	metE	38.5/52.0	5.2/5.1	6.1	42
E4T3S1	Glucose-1-phosphate thymidylyltransferase		32.5/58.0	5.4/5.2	8.0	46, 47
G8LHP0	Phosphoserine aminotransferase	serC	39.8/96.0	5.5/5.6	2.8	58, 50, 52, 60
G8LHN6	Formate acetyltransferase 1		7.5/98.0	6.5/5.2	5.7	5, 58
G8LK61	Aspartate carbamoyltransferase		34.4/90.0	5.7/5.2	15.2	58
E0SJV6	Glucose-1-phosphate adenylyltransferase	glgC	47.998.0	6.4/5.2	5.8	58, 60
D6RSA0	Fucosyltransferase FucT		15.8/40.0	9.4/5.3	8.9	29
B1YIN4	3-oxoacyl-(Acyl-carrier-protein) reductase		22.9/52.0	4.7/5.1	7.0	42
G8LHR1	Aspartate aminotransferase	aspC	43.3/98.0	5.5/5.2	14.9	58-60
U3N6X0	Chloroplast ATP-dependent zinc metalloprotease		12.8/62.0	6.3/4.7	27.7	21, 43

G0BPH2	2-hydroxypropyl-CoM lyase		38.8/60.0	5.2/5.5	23.6	46, 48
Q6TQD8	Beta-1,3-glucanase, basic	bgl	13.1/56.0	9.7/5.3	18.5	47
F6H740	Beta-D-xylosidase 1-like		83.2/98.0	8.4/5.2	16.8	58
D4GHI3	Beta-galactosidase (Beta-gal)		116.5/78.0	5.3/5.1	12.7	56-60, 50-54
P05457	Glutamine synthetase 1		52.3/58.0	5.4/5.0	3.2	45- 48, 58, 59
A5GZ76	Pyrroline-5-carboxylate synthetase	P5CS	76.9/26.5	6.6/4.3	10.6	13, 24
G8LF37	2,3-bisphosphoglycerate-dependent phosphoglycerate mutase		29.8/96.0	5.4/5.6	12.6	2, 58, 59, 60
E0SAX7	Cysteine desulfurase		44.9/98.0	5.7/5.2	5.0	58
D4GGI2	Tryptophanase	tnaA	53.0/96.0	6.3/5.0	11.4	1, 5, 56, 57, 60, 50, 51, 54, 53
Q304Q8	Catalase		25.7/78.0	6.7/4.6	4.9	54, 58
Q84R16	Exostosin family protein		60.9/28.0	5.7/4.7	6.1	14
B5LAS6	Putative acetyl co-enzyme A carboxylase		71.5/52.0	6.3/5.3	16.5	41
D7U1W8	Putative uncharacterized protein		30.5/20.0	6.35	10.3	3
D7U7F7	Putative uncharacterized protein		12.3/29.0	8.8/5.2	10.7	16, 59
C6SW43	Uncharacterized protein		13.9/13.0	9.5	16.4	1
**Signal transduction**						
P05132	cAMP-dependent protein kinase catalytic subunit alpha	Prkaca	40.6/20.0	8.84	20.2	1-5
Q67XP8	Calcium-dependent lipid-binding domain-containing protein		78.1/28.0	8.5/4.7	2.7	14, 16, 20, 21
B9T038	Calcium-binding protein, putative		13.3/96.0	9.0/5.0	14.4	56, 57
E3E6Z6	Adenylate kinase	adk	23.6/58.0	5.1/5.0	10.3	45
Q08480	Adenylate kinase B	ADK-B	79.9/30.0	6.02	4.5	5
E0SI39	Aspartokinase		48.9/98.0	5.1/5.2	8.4	58
B9HYQ3	Serine/threonine-protein kinase		91.8/31.0	7.3/5.2	10.5	20
F4JDN8	Protein kinase family protein		41.7/56.0	6.1/5.3	2.4	47
Q38919	Rac-like GTP-binding protein	ARAC4	21.6/46.0	9.3/4.8	4.6	36
D7U9Z6	Probable protein phosphatase 2C 5		31.3/40.0	7.6/5.3	2.8	29
B1Q489	Putative phosphate-induced protein		32.4/98.0	9.4/5.2	30.1	58
G3CHK6	Leucine-rich repeat family protein		41.74/98.0	9.2/5.2	7.8	58
**E0SF13**	**Methyl-accepting chemotaxis protein**		72.6/16.5	5.2/4.2	2.5	6, 11, 16
C6JT24	Gibberellin induced protein		11.8/26.5	8.9/4.3	15.2	13
Q5IVZ8	Phytosulfokine	PSK	8.6/25.0	6.3/5.2	15.6	17

Defense and stress responsive

B9SIF5	wound induced protein		10.0/13.0	9.76	24.4	1
I1N627	Hypersensitive-induced response protein 1-like		31.2/30.0	5.28	19.95	5
A7PQW3	Glucan endo-1, 3-beta-glucosidase		36.4/56.0	5.5/5.3	10.2	47
E2GEW7	Pathogenesis-related protein 1	PR-1	17.6/56.0	9.2/5.3	14.2	47
P32765	Miraculin-like, partial		16.2/40.0	5.6/4.3	6.8	33
Q93VR4	MLP-like protein 423	MLP423	17.1/42.0	5.1/5.3	9	17, 28
Q4ZYH6	AAA ATPase		95.2/72.0	5.4/5.9	11.9	49
A8HSN3	AAA ATPase		96.6/58.0	5.6/5.0	28.1	45, 48
E0SJW0	Cold shock protein		7.5/98.0	6.5/5.2	27.1	1, 2, 5, 50, 58-60
**P0AFH9**	**Osmotically-inducible protein Y**	osmY	21.1/13.0	6.32	20.9	1, 2, 5
**A2PZE5**	**CASP-like protein IN26**	IN26	20.1/27.0	9.57	5.9	4
B9RR27	Pectinesterase		46.898.0	9.1/5.2	20.1	58
Q94KI0	Mitochondrial processing peptidase		50.0/98.0	5.6/5.2	13.6	50, 58, 60, 61
E0XN34	Alpha-mannosidase		62.6/98.0	8.9/5.2	5.2	58
A5HIJ6	Cysteine protease	Cp6	51.8/98.0	5.3/5.2	3.4	58
P25076	Cytochrome c1-1, heme protein	CYCL	34.8/30.0	6.16	5.4	5
C5YZX1	NADH Cytochrome B5 reductase 1 isoform		27.1/24.0	9.2/4.8	10.8	7
Q6IV17	Protein disulfide isomerase		56.2/99.0	4.8/4.7	42.1	55
A9CHR5	NAD(P) transhydrogenase subunit alpha		55.9/78.0	5.7/4.6	1.5	51, 54
B3PDW0	NAD (FAD)-utilizing dehydrogenase		45.7/48.0	7.0/4.7	5.5	35
B9SGX5	Aspartic proteinase nepenthesin-2, putative		48.1/98.0	5.6/5.2	12.5	58
D7MCP0	Acid phosphatase class B family protein		26.1/96.0	6.0/5.4	5.4	58, 59
G8LEG4	Aldehyde-alcohol dehydrogenase	adhE	99.0/98.0	6.4/5.2	3.5	58
P0AF95	Enamine/imine deaminase		13.6/17.0	5.36	7	2
B9S114	Katanin p60 ATPase-containing subunit, putative		16.8/96.0	4.6/4.9	2.7	56
P0AE11	Alkyl hydroperoxide reductase subunit	ahpC	20.7/13.0	5.03	28.9	1, 2, 5
P0A866	Thiol peroxidase	tpx	17.8/13.0	4.75	13.1	1, 2, 5
P0C0L3	Peroxiredoxin OsmC	osmC	15.1/17.0	5.57	7	1, 2
A7NY33	Peroxidase 4		35.5/98.0	9.6/5.2	24.9	58
I2AWN0	Polyphenol oxidase	PPO	64.7/98.0	7.7/5.2	17	58
P23321	Oxygen-evolving enhancer protein 1-1	PSBO	35.1/58.0	5.5/5.2	31.3	5, 9, 22, 36, 46-49
Q8XB78	Molecular chaperone	Hsp31	31.1/13.0	5.54	5.3	1, 2, 5
P0AET4	Acid stress chaperone HdeB	hdeB	12.0/13.0	5.73	15.7	1, 2, 5

Q3K6N3	Chaperone	clpB	95.5/60.0	5.4/5.5	16.9	46, 48
Q4K5W1	ATP-dependent chaperone protein ClpB	clpB	95.0/56.0	5.2/5.3	16.7	47
A8I5R5	60 kDa chaperonin 2	groL2	57.3/58.0	5.1/5.0	29.0	44, 45, 47, 48
G0BJL5	60 kDa chaperonin	groL	57.4/98.0	4.9/5.2	13.0	1, 2, 5, 42, 46, 49, 57- 60
G8LIG0	10 kDa chaperonin	groS	14.1/98.0	5.9/5.2	27.1	1, 2, 5, 45, 47, 48, 50, 58-60
P94317	Chaperone protein DnaK (HSP70)	dnaK	68.2/58.0	5.1/5.0	20.5	1, 2, 5, 45, 46, 48, 59, 60
E0SD70	ATP-dependent Clp protease	ClpX	46.5/58.0	5.4/5.0	13.4	45, 46
B3PDZ1	ClpB	clpB	99.6/58.0	5.5/4.9	8.4	23, 42, 44, 47- 49
Q71U34	Heat shock cognate 71 kDa protein	HSPA8	70.9/20.0	5.37	6.8	3
Q4LDR0	Heat shock protein	ClpB	90.1/60.0	5.4/5.5	19.2	23, 45-48
F6HW56	heat shock 70 kDa protein		72.9/60.0	5.8/5.5	10.0	3, 4, 45, 48
Q9ZT13	101 kDa heat shock protein	HSP101	100.7/56.0	5.9/5.3	13.3	43, 44, 46, 47
G8LET4	Small heat shock protein ibpA	ibpA	15.7/98.0	5.4/5.2	14.0	58
D8IT51	ATP-dependent Clp protease subunit (Heat-shock) protein	clpB	95.0/60.0	5.5/5.5	19.5	48
G8LL06	Methylenetetrahydrofolate reductase	metF	33.2/98.0	6.3/5.2	4.4	58
Q87U42	Rubredoxin reductase	rub	40.2/20.0	5.4/4.1	4.2	11
D4GIG2	AhpC	ahpC	20.6/98.0	5.0/5.2	26.2	58
P0ADU6	Protein YgiW	ygiW	14.0/13.0	5.08	6.9	1
F4JD33	Phloem protein 2-A13	PP2-A13	33.2/46.0	8.7/4.8	3.1	13, 36
B9T876	Minor allergen		21.8/42.0	6.4/5.9	22.2	27
B1Q4T6	Uncharacterized protein		62.8/18.5	6.4/5.3	13.1	9
Q9FPD1	Putative uncharacterized protein		22.1/29.0	7.6/5.0	35.2	15
**Membrane trafficking**						
F5C0G9	SNAP33	SNAP33	12.2/56.0	6.9/5.3	20.2	47
Q4W5U7	Calnexin-like protein		61.1/23.0	4.6/4.9	7.3	8
F6I279	Putative uncharacterized protein		63.9/78.0	5.1/4.6	2.0	54
**Unchanracterized**						
D7SHW9	Putative uncharacterized protein		10.8/17.0	10.39	10.5	2
Q84WV3	Putative uncharacterized protein		27.4/42.0	8.3/4.8	3.2	31
Q9ZUX9	Putative uncharacterized protein		42.3/31.0	9.8/6.2	3.3	18
B9S140	Putative uncharacterized protein		11.2/32.0	4.9/5.3	22.3	12, 19, 22, 27
B1Q4U0	Uncharacterized protein		26.4/62.0	7.6/4.7	5.9	43
Q4KAX0	Uncharacterized protein		38.1/58.0	5.7/5.2	6.4	46

E0SEA2	Uncharacterized protein		38.0/58.0	5.9/5.0	7.3	45, 47
Q8GEG0	Uncharacterized protein		14.3/96.0	6.3/5.0	10.6	53, 56, 57
Q4ZFU4	Uncharacterized protein		16.0/48.0	4.7/4.7	8.0	35
Q66GQ2	Uncharacterized protein		70.8/46.0	8.9/4.8	3.2	36
B9HFF7	Uncharacterized protein		38.3/42.0	6.3/4.8	4.4	31
I1LJG7	Uncharacterized protein		18.1/42.0	4.7/5.2	5.0	30
Q0RF97	Uncharacterized protein		38.8/52.0	10.9/5.3	5.6	41
**Probable contaminants**						
Q9BXS6	Nucleolar and spindle-associated protein 1	NUSAP1	49.4/20.0	9.92	33.3	3, 4
Q93131	Actin, cytoplasmic		41.7/20.0	5.3	22.7	3, 4
Q9FWA6	Pentatricopeptide repeat-containing protein		101.7/28.0	5.9/4.7	4.7	14
Q9SZL8	Protein FAR1-RELATED SEQUENCE 5	FRS5	90.4/28.0	6.4/4.7	1.4	14
Q7CTQ7	Transcriptional regulator, GntR family		25.8/42.0	6.3/5.3	5.2	14
G9FCF4	ATP synthase subunit beta, chloroplastic	atpB	53.8/78.0	5.1/4.9	31.3	34, 35, 47, 52, 56, 30, 41
Q32AF8	50S ribosomal protein L7/L12	rplL	12.3/13.0	4.6	25.6	1, 2, 50, 52
B3TLN9	Cytoplasmic ribosomal protein		14.8/17.0	9.89	13.1	2
G8LHF3	30S ribosomal protein S3	rpsC	25.9/30.0	10.27	8.2	5, 48
Q2JFI0	30S ribosomal protein S7	rpsG	17.2/58.0	10.4/5.2	26.3	45-49
B3PK47	50S ribosomal protein L14		13.4/58.0	10.2/5.0	38.5	5, 45, 46, 49
G8LI39	50S ribosomal protein L2	rplB	29.8/96.0	10.9/5.6	14.3	54, 57, 59- 61
G8LHP3	30S ribosomal protein S1	rpsA	61.2/98.0	4.9/5.2	13.6	44, 58, 59, 60
D4GNY3	50S ribosomal protein L9	rplI	15.7/98.0	6.7/5.2	47.7	5, 58
A9XP58	40S ribosomal protein S14		12.3/18.5	10.0/5.3	18.1	9
G9IB70	Ribulose bisphosphate carboxylase large chain	rbcL	52.8/38.0	6.3/5.4	26.2	47, 54, 57-59, 21, 14-16
G9FCF5	Ribulose bisphosphate carboxylase large chain	rbcL	54.5/56.0	6.1/5.3	14.2	2, 13, 20, 23, 24, 47, 54
I0B7J4	Chloroplast PsbO4	psbO4	35.0/36.0	5.3/4.5	33.5	5, 8, 9, 22, 45, 46
B9SID9	Chlorophyll A/B binding protein, putative		49.4/27.0	9.92	13.6	4, 5, 37, 47
A5AE63	chlorophyll a-b binding protein 6A		26.6/20.0	5.4	8.6	3, 4, 32, 36, 30
Q84QE7	Putative photosystem I subunit III		28.6/27.0	5.33	14.0	2, 3, 4
G7JG11	Photosystem II D2 protein		39.8/56.0	5.3/5.3	11	4, 5, 47
Q0PWS7	Chloroplast pigment-binding protein CP29		43.4/27.0	6.02	10.3	4
P12372	Photosystem I reaction center subunit II	psaD	22.6/20.0	9.73	15.1	3
B9SMR5	Photosystem I reaction center subunit XI		23.1/17.0	9.39	11.9	2
F4YFK3	Photosystem I reaction center V		18.7/13.0	9.4	15.1	1, 5

B9R8G0	Plastocyanin A		16.8/13.0	4.9	33.1	1, 1
H2B849	Photosystem II	psbC	51.8/96.0	6.6/5.4	6.3	1, 2, 58, 59
E1SBX6	Alanyl-tRNA synthetase	alaS	96.2/16.5	5.3/4.2	1.1	6
E0RGP1	DNA-directed RNA polymerase subunit beta		132.8/58.0	5.1/5.0	7.5	23, 44, 45, 48, 49, 60
Q8LE51	Protein yippee-like		14.5/42.0	7.7/5.9	17.1	27
G8LI77	Catabolite gene activator		23.6/98.0	8.4/5.2	3.8	58
B9VH85	Photosystem Q (B) protein	psbA	38.8/56.0	5.2/5.3	9.1	5, 47
Q5FQL9	Polyribonucleotide nucleotidyltransferase	pnp	77.2/60.0	5.5/5.5	11.9	45- 48
G8LI48	Elongation factor Tu (EF-Tu)	tuf1	43.3/98.0	5.2/5.2	48.2	5, 34, 44, 45, 48, 56-60, 50-54
Q84RU1	Elongation factor 1-alpha	EF1-A	29.1/27.0	6.02	12.9	1-4
E1SAT8	Integration host factor subunit alpha		9.5/13.0	9.57	25.6	1, 5
Q9CAX7	Putative small nuclear ribonucleoprotein		8.7/13.0	8.06	11.4	1
Q03684	Luminal-binding protein 4 (BiP 4)	BIP4	28.1/30.0	8.45	10.3	3, 5
Q03685	Luminal-binding protein 5 (BiP 5)	BIP5	73.4/78.0	5.1/4.6	7.8	2, 54
Q9U7D0	Histone H4		11.9/20.0	11.48	16.7	3, 58
P42740	Polyubiquitin		51.5/27.0	7.05	11.8	4
F6HI72	Putative chlorophyllase-1		37.1/20.0	8.09	7	3
P65976	Protein RecA (Recombinase A)	recA	38.7/60.0	5.2/5.5	9.1	48

Supplementary Table 2:

Tonoplast proteins identified by Mass spectrometry

Proteins are grouped according to functional similarity. GO annotation was carried out using the STRAP software (http://www.bumc.bu.edu/cardiovascularproteomics/cpctools/strap/). Protein ID is given in column 1. Together with commonly used abbreviations, if available, the protein names are given as in uniprot/ (STRAP) in column 2 and 3. In column 4 and 5, the theoretical and experimental (Thr/Exp) molecular weight and p*I* respectively, are given. The molecular weight and p*I* were calculated using ExPASy (http://web.expasy.org/compute_pi/). The % coverage is given in 6 and band / spot numbers are indicated in the column 7.

Protein ID	Protein description	protein name	MW (kDa) Thr/Exp	p*I* Thr/Exp	%cov	band/spot index	
**Transport**							
P09469	V-type proton ATPase catalytic subunit A		68.6/19.0	5.3/6.8	0	8.2	69, 82
F4JTQ0	V-type proton ATPase subunit B2		10.7/88.0	6.5/4.9	0	32.6	68, 87, 88
Q8W4E2	V-type proton ATPase subunit B3	VHA-B3	54.3/78.0	4.9/5.1	0	42.3	86
Q0WWD3	Vacuolar-type H+-ATPase subunit D		29.1/30.0	9.52	0	13.0	66
Q9LJI5	V-type proton ATPase subunit d1	VHA-D	40.8/70.0	5.04	0	2.3	68
Q41396	V-type proton ATPase subunit E	VATE	26.4/30.0	6.85	0	7.9	66, 67
B7FMK2	V-type proton ATPase subunit E1		26.5/30.0	6.52	0	10.9	66, 68
A5B4N7	V-type proton ATPase subunit F		14.5/20.0	7.99	0	29.5	63
O82702	V-type proton ATPase subunit G 1	VATG1	12.1/20.0	6.84	0	8.2	63, 67, 68
F1CFB4	Vacuolar ATP synthase subunit		26.3/28.0	8.71	0	10.1	65- 67
B9SAL7	ATP synthase subunit d		29.1/30.0	9.58	0	18.8	66, 72
B9R8F3	ATP synthase subunit beta vacuolar		12.3/70.0	4.87	0	50.4	68, 86, 87
B9S5J4	ATP synthase subunit beta vacuolar		23.7/88.0	4.8/4.9	0	42.2	88
K4KD26	ATP synthase subunit beta	atpB	54.0/44.0	5.3/5.0	0	6.2	83
Q9CKW2	ATP synthase subunit alpha	atpA	55.7/70.0	5.24	0	3.7	68
Q4QX18	Vacuolar ATP synthase		16.7/70.0	4.59	0	19.9	68
B9HRF1	Vacuolar ATP synthase		12.9/20.0	9.30	3	22.5	63

D7STX7	Cation transporting ATPase		25.8/32.0	9.4/4.8	0	4.1	77
G9JV85	AVP1-1		7.4/70.0	3.95	0	15.1	68
G0BKG0	Inorganic pyrophosphatase	ppa	19.6/20.0	5.09	0	10.2	63, 68
Q0RBI5	K+-insensitive pyrophosphate-						
	energized proton pump	hppA	75.8/70.0	5.58	15	4.0	68
Q898Q9	Putative K+-stimulated pyrophosphate-						
	energized sodium pump (Na+-PPase)	hppA	69.2/70.0	5.27	15	3.1	68
A9CTP9	Tonoplast intrinsic protein	TIP1-1	25.6/20.0	6.2	7	4.1	63, 64, 66- 68
Q537C0	Aquaporin 1	PIP1	30.5/70.0	8.86	6	15.3	67, 68
Q6DLS9	Plasma membrane intrinsic protein	PIP1-1	36.4/70.0	9.24	7	9.2	64, 66, 68
Q8LAA6	Probable aquaporin PIP1-5	PIP1-5	30.6/70.0	8.99	6	10.8	66, 67, 68
A1YS44	Aquaporin 2	PIP2	29.8/42.0	9.14	6	4.3	67
Q8W1A8	Aquaporin-like protein	PIP2-1	10.8/30.0	4.53	2	13.3	66, 67, 68
B8Q9D4	Aquaporin PIP2-4	PIP2-4	9.2/42.0	9.4	2	26.7	67, 68
Q9XF58	Aquaporin PIP2-5	PIP2-5	29.8/70.0	7.7	6	4.2	67, 68
Q9SV31	Probable aquaporin PIP2-5	PIP2-5	30.6/70.0	8.99	6	4.2	64, 68
O23771	Major intrinsic protein PIPB	PIPb	31.0/70.0	8.99	6	8.7	67, 68
Q7D317	ABC transporter, membrane spanning protein		86.9/19.0	10.1/5.2	11	1.4	71
E1SJC2	ABC-type sugar transport system,	rbsB	30.5/16.0	8.61	0	29.2	62, 63, 64, 66, 68
E0SN93	Urea carboxylase-related ABC transporter,		31.4/32.0	6.7/4.8	0	4.2	77
K4KCZ8	Ycf1 (Fragment)	ycf1	78.8/25.0	9.5/5.2	6	5.4	72a
P02925 76, 84, 89	D-ribose-binding periplasmic protein	rbsB	30.9/16.0	6.85	0	43.2	62, 66, 68, 69, 72,
P0AEE7	D-galactose-binding						
	periplasmic protein	mglB	35.7/20.0	5.68	1	8.1	62-64, 66, 68
G8LMD4	Glucose-specific phosphotransferase						
	enzyme IIA component	crr E	18.2/16.0	4.73	0	16.6	62- 64, 66
B9R7V2	Sugar transporter, putative		9.8/30.0	8.61	1	11.1	66, 67
Q8LSZ0	Betaine/proline transporter		48.0/32.0	7.2/4.8	11	6.6	77
G8LJK7	Acyl carrier protein (ACP)	acpP	86.1/16.0	3.98	0	32.1	62, 63, 64, 66, 68
D7MMV7	Band 7 family protein		16.4/30.0	5.42	0	33.6	66
Q38JD4	Temperature-induced lipocalin	TIL	21.3/44.0	6.2/5.0	0	15.7	83
Q9AXF3	Non-specific lipid-transfer protein		11.7/30.0	8.99	0	10.3	66

Metabolic process

G8LLY7	Transaldolase	tal	35.2/20.0	5.0	2.8	63, 64, 68
B9GZ15	Transaldolase family protein		9.7/19.0	4.8/6.8	12.6	69, 72, 76, 83, 89
P0A958	KHG/KDPG aldolase		22.3/20.0	5.6	8.9	63, 68
Q9LYR4	Aldolase-type TIM barrel family protein		47.6/19.0	6.1/6.8	2.5	69, 73, 76, 83, 89
P0AB73	Fructose-bisphosphate aldolase class 2	fbaA	39.1/20.0	5.5	4.7	62, 63, 66, 68
Q3YX11	Malate dehydrogenase	mdh	3.2/16.0	5.6	21.2	62-64, 66, 68, 69, 72, 76
A9XP12	Malate dehydrogenase (NADPH)		13.1/62.0	4.7/5.7	9.2	85
Q9XQP4	NAD-malate dehydrogenase		43.5/26.0	7.0	10.7	64, 68, 72
P25553	Lactaldehyde dehydrogenase	aldA	52.3/16.0	5.1	5.4	62-64, 66, 68
D8HNM0	NAD-dependent aldehyde dehydrogenase		51.9/16.0	5.1	1.6	62, 63
G8LKG3	Glyceraldehyde-3-phosphate dehydrogenase A	gapA	35.9/20.0	6.3	12.3	62-64, 66, 67
P08200	Isocitrate dehydrogenase [NADP]	icd	45.7/20.0	5.1	7.5	62-64, 66, 68
G8LD75	2-dehydro-3-deoxy-D-gluconate 5-dehydrogenase		27.2/16.0	5.4	7.9	62-64, 66, 68
D5BM76	Glycine dehydrogenase (decarboxylating)		104.3/30.0	5.5	1.4	66
G0BLU1	Delta-1-pyrroline-5-carboxylate dehydrogenase	SerA	144.4/20.0	5.5/5.1	1.9	72
A8IJK7	Putative succinate-semialdehyde dehydrogenase		53.1/27.0	5.7/5.3	3.0	76, 83
Q7UB84	Glycerol kinase	glpK	56.2/16.0	5.3	21.3	62-64, 66, 68
G8LIA9	Phosphoenolpyruvate carboxykinase	pckA	59.7/20.0	5.4	1.9	63, 64
Q8XD03	Phosphoglycerate kinase	pgk	41.1/26.0	5.1	10.3	62-64, 68, 76
G0BP26	Enolase	eno	45.4/26.0	5.23	14.6	62-64, 66, 68
G8LF07	Citrate synthase		4.8/16.0	5.9	2.6	62-64, 66, 68
P05457	Glutamine synthetase 1	glnA	52.3/19.0	5.4/6.8	3.2	69, 73, 86
A5GZ76	Pyrroline-5-carboxylate synthetase	P5CS	76.9/20.0	6.6/4.7	9.9	73, 85
D4GGI2	Tryptophanase	tnaA	53.0/16.0	6.3	34.2	62-64, 66, 68, 76, 69, 83
G8LF15	Succinyl-CoA ligase subunit alpha	sucD	29.8/16.0	6.1	7.6	62-64, 66, 68
Q8YMW8	Serine hydroxymethyltransferase (SHMT)		45.4/26.0	5.2	14.6	64
Q4ZXZ0	Glucose-1-phosphate thymidylyltransferase		32.3/19.0	5.4/6.8	6.1	69, 72, 83
B3PIH6	UTP-glucose-1-phosphate uridylyltransferase		31.5/20.0	5.8/5.1	6.4	72
A9CFQ4	5-methyltetrahydropteroyltriglutamate -homocysteine methyltransferase	metE	38.7/20.0	5.6/5.1	21.6	72, 89
F4IV00	Phosphatidylinositol N-acetylglucosaminyl transferase subunit P-like protein		52.9/44.0	6.7/5.2	1.9	84a
D6RSA0	Fucosyltransferase	FucT	15.8/25.0	9.4/5.2	8.9	72a
Q83PR1	Glutamate decarboxylase alpha (GAD-alpha)	gadA	52.7/26.0	5.2	18.9	62-64, 66, 68

B8Q219	UDP-glucose pyrophosphorylase	ugp	50.9/70.0	6.4	1.9	68
Q4KAW9	Methionine synthase family protein, vitamin-B12 independent		38.4/19.0	5.3/6.8	15.5	69, 72, 83, 89
D4GHI3	Beta-galactosidase (Beta-gal)		116.5/20.0	5.3/5.1	3.8	72, 76
G0BPH2	2-hydroxypropyl-CoM lyase	SerA	38.8/90.0	5.2/5.5	13.1	91
P48495	Triosephosphate isomerase	TPIP1	27.0/90.0	5.9/5.5	34.3	91
Q7XBZ2	3-hydroxyisobutyryl-coenzyme A hydrolase		40.7/78.0	10.8/5.0	25.5	87
B1YFD1	Peptidyl-prolyl cis-trans isomerase		39.7/78.0	4.7/5.0	5.3	65, 67, 87, 90
G8LID3	Ribose 1,5-bisphosphate phosphokinase	PhnN	20.5/36.0	9.3/5.1	6.0	83
B5LAS6	Putative acetyl co-enzyme A carboxylase		71.5/35.0	6.3/4.8	21.4	79, 84
**Signal transduction**						
F4KBI7	14-3-3-like protein GF14	GRF3	28.5/30.0	4.8	3.2	66
A1Y295	Calcium-dependent protein kinase	CDPK	14.9/32.0	9.7/4.8	11.0	77
P05132	cAMP-dependent protein kinase catalytic subunit alpha (PKA C-alpha)	Prkaca	40.4/42.0	8.85	20.2	62-64, 66-68
Q67XP8	Calcium-dependent lipid-binding domain-containing protein		78.1/18.0	.5/6.2	1.4	70, 88, 90
**D4IF83**	**Methyl-accepting chemotaxis protein**		57.4/19.0	5.8/5.2	1.7	71
D7MCP0	Acid phosphatase class B family protein		26.1/90.0	6.0/5.5	5.4	91
B9S9I9	Magnesium-dependent phosphatase, putative		21.5/78.0	8.5/5.1	10.2	86
Q2JFT4	Ppx/GppA phosphatase		33.3/78.0	5.8/4.8	6.1	90
B1YLZ1	Diguanylate cyclase/phosphodiesterase		107.6/20.0	5.34	0.9	63
Q87W18	Uncharacterized protein		75.6/18.0	9.8/6.2	1.3	70
**Defense and stress responsive**						
Q9SRH6	Hypersensitive-induced response protein 3	HIR3	31.2/30.0	5.67	15.8	66-68
I1N627	Hypersensitive-induced response protein 1		31.2/30.0	5.28	28.3	66, 67, 76
Q672Q3	Wound/stress protein		19.6/27.0	4.7/5.3	35.0	76
K4KF95	Rps4	rps4	24.5/78.0	10.6/4.9	4.3	88
Q0RFY7	Tellurium resistance protein		16.3/20.0	5.18	6.0	63
Q93VR4	MLP-like protein 423	MLP423	17.1/44.0	5.1/5.2	9.0	84a
A8HSN3	AAA ATPase		96.6/20.0	5.7/5.1	3.5	72
G8LKX5	Thioredoxin	trxA	12.4/70.0	4.59	13.3	68
**A2PZE5**	**CASP-like protein IN26**	IN26	20.1/30.0	9.57	5.9	66
F4YF81	Cytochrome P450		14.5/19.0	8.9/6.8	18.3	69

Q9AXH1	Glyoxalase I		20.7/44.0	5.2/5.0	14.1	83
**G8LL62**	**Osmotically-inducible protein Y**	osmY	22.4/16.0	8.92	19.6	62-64, 66, 68
G8LG88	Cold shock-like protein	CspC	7.7/20.0	6.54	31	62-64, 66, 68
**P0C0L3**	**Osmotically inducible protein C**	osmC	15.1/26.0	5.57	7.0	62, 64, 68
G8LLZ2	Chaperone protein DnaK (HSP70)	dnaK	69.1/16.0	4.82	6.6	62-64, 66, 68, 83
G8LIG1	60 kDa chaperonin (GroEL protein)	groL	57.1/16.0	4.85	22.3	62-64, 66, 68
A8I5R5	60 kDa chaperonin 2	groEL2	57.3/19.0	5.1/6.8	12.2	69, 76, 83
I7GVS5	Heat shock protein 70	Hsp70	71.1/42.0	5.1	5.1	62, 65, 67, 83
Q8XB78	Molecular chaperone Hsp31 and glyoxalase 3		31.2/16.0	5.54	5.3	62-64, 66, 68
Q3YUJ8	10 kDa chaperonin (GroES protein)	groS	10.4/20.0	5.15	42.3	62-64, 66, 68, 72
Q9ZT13	101 kDa heat shock protein	HSP101	100.7/19.0	5.9/6.8	20.3	69, 72
Q4LDR0	Heat shock protein	ClpB	109.5/44.0	6.0/5.0	9.3	83
P0AET4	Acid stress chaperone HdeB	hdeB	12.0/16.0	5.73	15.7	62-64, 66, 68
B3PDZ1	ClpB	clpB	99.6/19.0	5.5/6.8	8.7	69 76, 83
D8IT51	ATP-dependent Clp protease subunit	clpB	95.0/20.0	5.5/5.1	10	69, 72, 76, 83
P0A866	Thiol peroxidase	tpx	17.8/16.0	4.75	13.1	62-64, 66, 68
Q87U42	Rubredoxin reductase	rubB	40.2/32.0	5.4/4.8	4.2	77
P0AE11	Alkyl hydroperoxide reductase subunit C	ahpC	20.8/20.0	5.03	28.9	62-64, 66, 68, 69, 72, 76, 89
Q8VX72	Putative cysteine proteinase inhibitor		12.9/20.0	9.3/5.1	44.7	72
Q42891	Lactoylglutathione lyase	GLX1	20.7/44.0	5.2/5.0	5.4	83
A9XP75	Acireductone dioxygenase		16.2/25.0	4.8/5.2	7.4	72a
S8E5Q8	Mitochondrial processing peptidase		58.7/35.0	6.1/4.7	12.9	78
A5C961	Transmembrane ascorbate ferrireductase 1		25.0/20.0	7.05	9.1	63, 66, 68
Q9LRC4	Oxygen evolving enhancer protein 1	psbO	35.1/78.0	6.5/4.9	10.2	88
A6UD50	Sarcosine oxidase, alpha subunit family		107.4/30.0	6.44	0.8	63, 66
B1Q4T6	Uncharacterized protein		62.8/35.0	6.4/4.7	5.6	78
**Membrane trafficking**						
B9SNS6	Syntaxin, plant, putative		29.7/30.0	6.26	5.9	66
F5C0G9	SNAP33	SNAP33	25.5/20.0	6.2/5.1	9.2	72
B9RTN6	Lipid binding protein, putative		10.3/78.0	8.5/4.9	8.1	69
**Uncharacterized**						
A5BPL0	Putative uncharacterized protein		7.5/19.0	5.2/6.8	17.4	69
A5C8A4	Putative uncharacterized protein		15.0/78.0	9.2/5.1	11.2	86
B9SNE9	Putative uncharacterized protein		39.3/78.0	9.8/4.9	3.6	89

Q9C7L8	Putative uncharacterized protein		10.5/26.0	9.4/5.1	9.4	75
A5AZL6	Putative uncharacterized protein		30.0/19.0	5.0/4.1	8.2	74
B9S140	Putative uncharacterized protein		11.2/35.0	4.9/4.8	11.6	79
D7STS6	Putative uncharacterized protein		55.6/44.0	8.4/5.0	5.3	84
F4IQ39	Uncharacterized protein		75.8/32.0	5.2/4.8	7.8	77
F4ING4	Uncharacterized protein		142.6/26.0	10.1/5.1	5.7	75
**Probable contaminants**						
Q9Y707	Actin-2	ACT2	41.7/28.0	5.31	6.7	65
P0ACF3	DNA-binding protein HU-alpha	hupA	9.5/16.0	9.57	25.6	62-64
Q9SZL8	Protein FAR1-RELATED SEQUENCE 5		90.1/26.0	6.4/5.1	3.8	75
G8LHP3	30S ribosomal protein S1	rpsA	61.2/16.0	4.9	4.7	62-64, 66, 68
E0SGF1	50S ribosomal protein L7/L12	rplL	12.4/16.0	4.6	25.4	62-64, 66, 68, 83
Q8FZE8	50S ribosomal protein L25	rplY	22.4/19.0	5.9/6.8	3.9	69
Q2JFI0	30S ribosomal protein S7	rpsG	17.2/19.0	10.5/6.8	18.0	69, 72, 76, 83
G8LI49	Elongation factor G (EF-G)	fusA	77.6/26.0	5.2	1.6	64
Q84RU1	Elongation factor 1-alpha	EF1-A	36.3/28.0	9.2	8.4	65, 67
G8LI48	Elongation factor Tu (EF-Tu)	tuf1	43.3/16.0	5.3	31.7	62, 63, 66, 68, 69, 72, 74, 83
B9T0C1	Chlorophyll A/B binding protein, putative		28.7/26.0	5.5	4.5	64
G9IB70	Ribulose bisphosphate carboxylase large chain	rbcL	52.9/70.0	6.3	14.7	68, 72, 76, 89, 83
A9XP95	Ribulose bisphosphate carboxylase small chain		20.2/20.0	9.1/4.7	19.1	63, 73, 87
B9R8G0	Plastocyanin A, chloroplast, putative		16.8/19.0	4.9/6.8	49.4	69
Q5FQL9	Polyribonucleotide nucleotidyltransferase	pnp	77.2/27.0	5.5/5.3	8.0	69, 76, 83
Q03685	Luminal-binding protein 5 (BiP 5)	BIP5	73.4/70.0	5.14	5.9	68
D4ICJ8	ATP-dependent DNA helicase		68.5/20.0	7.88	1.5	63
E1SAT8	Integration host factor subunit alpha (IHF-alpha)	hupa3	9.5/16.0	9.57	25.6	62, 63, 66, 68
P42740	Polyubiquitin		51.5/28.0	7.05	11.8	65
C9YZF6	Putative two-component system response regulator		26.9/32.0	5.5/4.8	7.9	77
Q04347	Bud site selection protein 22	BUD22	60.1/70.0	8.86	1.7	68

Supplementary Table 3:

INDEX to Spot/band numbering and PRIDE numbering: The band/spot numbers for the plasma membrane proteins are shown in column 1 and the corresponding index in PRIDE is shown in column 2. Similarly, the band/spot numbers for the tonoplast proteins are shown in column 3 and the corresponding index in PRIDE is shown in column 4.

**Plasma membrane**			**Tonoplast**	
band/spot number	Index in PRIDE		band/spot number	Index in PRIDE
1	P1		62	T1
2	P2		63	T2
3	P3		64	T3
4	P4		65	T4
5	P5		66	T5
6	P50		67	T6
7	P65		68	T7
8	P66		69	T1 (120510)
9	P67		70	T16

10	P68		71	T20
11	P59		72	T2 (120510)
12	P60		72a	T19
13	P58		73	T21
14	P57		74	T22
15	P56		75	T18
16	P55		76	T3 (120510)
17	P62		77	T26
18	P84		78	T24
19	P52		79	T25
20	P53		80	T27
21	P54		81	T29
22	P69		82	T28
23	spl13		83	T4 (120510)
24	P82		84	T34
25	P83		84a	T30
26	P51		85	T38
27	P89		86	T10
28	spl88		87	T11
29	P87		88	T12
30	P86		89	T13
31	P80		90	T14
32	P70		91	T5 (120510)
33	P71			
34	P73			
35	P75			
36	P79			
37	P81			
38	P76			
39	P77			
40	P78			
41	P90			
42	spl20			
43	spl21			
44	spl29			
45	spl26			
46	spl25			
47	spl24			
48	spl23			
49	spl22			
50	spl47			
51	spl44			
52	spl43			
53	spl42			

54	spl41			
55	P40			
56	spl39			
57	spl36			
58	spl35			
59	spl33			
60	PM30			
61	spl46			
